# Psychometric evaluation of nursing interns’ consciousness of rights scale in clinical practice

**DOI:** 10.1186/s12912-023-01622-0

**Published:** 2023-12-01

**Authors:** Yuting Zeng, Hongyu Li

**Affiliations:** Department of Nursing, Jinzhou Medical University, Jinzhou, China

**Keywords:** Nursing, Interns, Awareness of rights, Validation study

## Abstract

**Objective:**

The purpose of this study was to translate the Awareness of Rights Scale into Chinese and test its psychometric properties among nursing students in clinical practice.

**Methods:**

The original English scale was translated, synthesized, and back-translated according to the Brislin translation model: the translated scale was cross-culturally adapted through expert correspondence and pretesting to form the Chinese version of the scale; a convenience sampling method was used to survey 486 nursing interns in Liaoning, Guangdong, and Anhui regions to assess the reliability and validity of the scale.

**Results:**

The Chinese version of the scale consists of 14 items in three dimensions. The Cronbach’s alpha value of the scale was 0.916 and the range of Cronbach’s alpha coefficients of subscale was 0.768 to 0.894. The discounted half reliability was 0.867 and the retest reliability was 0.901. The scale content validity index (S-CVI) was 0.963. A total of three common factors were extracted for the exploratory factor analysis. The confirmatory factor analysis indices fit well (χ^2^/df = 1.092, RMSEA = 0.014, CFI = 0.998, IFI = 0.998. TLI = 0.997), and the model fit was good.

**Conclusion:**

The Chinese version of the scale has good reliability and validity in the nursing intern population and can be used to assess nursing interns’ awareness of their rights in clinical practice in mainland China.

## Background

In modern society, people’s legal awareness is increasing, and patients’ awareness of self-protection is becoming stronger. In hospitals, medical disputes and patients’ complaints are often caused by various reasons, and the personal rights of health care workers are often violated [1], so it is necessary to improve the rights awareness of health care workers. Rights awareness refers to people’s knowledge, understanding and attitude toward legal rights, people’s understanding of the content and types of rights, their choice of the way to realize rights, and their psychological reflection of consciously exercising rights and respecting others’ rights according to the law [2]. As nursing interns who are still students, they are even less aware of their rights and are unaware of the ways to ensure that their rights are upheld [3]. During hospital internships, the insufficient number of nursing staff leads nursing students to spend most of their time on patient care, and clinical instructors generally leave the less risky tasks to nursing students, with some specialist nursing operations with strong technical operations done by clinical instructors themselves, so that nursing students do not have the opportunity to practice specialist nursing operations [4], Therefore, the quality control of clinical training education by the school and the support students receive from their clinical instructors are important factors that affect the right to learn of nursing students [5]. Some patients in the clinical setting do not recognize nursing students as part of the nursing team and remain skeptical and distrustful of their abilities [6]. Campbe noted through a survey [7] that nursing students often feel vulnerable in the clinical setting and they are also in great need of respect, support, and recognition from clinical workers. However, due to historical reasons and objective conditions, nursing students are generally less aware of their rights in the clinical setting and lack the knowledge and ability to protect their rights as they should [8].

In this context, schools and hospital nursing departments should be aware that they serve as appropriate role models who should motivate nursing students and raise awareness of their rights [9]. This facilitates nursing students to complete high quality placements and plays an important role in enhancing their satisfaction with their placements and helping them to develop a good professional perception [10]. In addition, making the right clinical learning environment available at the right time gives nursing students the opportunity to complement theory with practice, improve their professional and technical skills [11], and increase nursing efficiency [12]. It also allows nursing students to increase their motivation for employment, while contributing to a better understanding of patients’ needs, relieving patients’ tension and anxiety, and helping to establish a good nurse-patient relationship with patients to provide better care for them [13].

Research on nursing students’ awareness of their rights in clinical nursing practice is limited, and there is no integrated scale for evaluating the consciousness of the students in the clinic related rights aspects. Instead, there are more scales evaluating the particular areas associated with patient care (such as introspection, pressure perception, work satisfaction).Professor Sunghee Park tested this idea with a mixed model created by Schwartz-Burcott and Kim, and used it to develop a scale that was confirmed and compiled in 2021 [14]. The research significance of the scale is that it lays the foundation for the first time for assessing nursing students’ awareness of their rights in a clinical setting, and it is able to comprehensively evaluate the level of nursing interns’ awareness of their rights from multiple perspectives.

This research aims at making a translation of the former English version into Mandarin, and evaluating the psychological characteristics of the English language in order to improve the understanding of the students’ rights. Therefore, it is believed that the Chinese edition of this questionnaire is reliable and effective, which can be taken as an instrument to evaluate the consciousness of righ the nursing students in China.

## Methods

### Study design and subjects

A total of 486 participants from Liaoning, Anhui and Guangdong provinces were recruited using a convenience sampling method in a multicenter cross-sectional survey conducted in China between December 2022 and February 2023. Participants were recruited from practicing nurses in three tertiary hospitals in Jinzhou, Hefei and Shenzhen. The required sample size was ≥ 3 subjects per item. In this study, a minimum of 20 participants per project was required to ensure the accuracy of the exploratory factor analysis and the confirmatory factor analysis [15]. The following criteria should be met for inclusion: (1) full-time fresh intern nurses; (2) bachelor or college degree; (3) clinical internship duration ≥ 6 months; (4) Informed consent and voluntary participation of nursing interns. Exclusion criteria: (1) intern nurses were not on duty during the survey period due to sickness or personal leave; (2) intern nurses were not involved in shift work.

### Instruments

The Clinical Nursing Trainee Rights Awareness Scale was developed by Sung-Hee Park Scholars et al. in 2021 for the measurement of trainee nurses’ rights awareness. The scale contains 14 entries divided into 3 dimensions: the right to be protected, care, support, and respect, the right to learn, and the privilege of being recognised as a member of the care group. The Likert Five Rating Scale was applied, with a rating between one and five for “totally disagree” to “totally agree”, and the total score range was 14–70 points, with higher scores indicating better awareness of the rights of student nurses.

### Procedures

The Cronbach’s alpha coefficient for the original scale was 0.92. After obtaining the authorization of the original authors, the scale was translated into Chinese and adapted using the Brislin double translation-back translation model [16]. (1) Forward translation: two master’s degree students in nursing who were native Chinese speakers and proficient in English translated the scale independently and obtained two first drafts of the translation. After comparison, discussion, and correction by the research group, a Chinese version was formed.1 ②Back translation: one PhD in nursing and one nursing expert proficient in English translated the first draft into English and compared it with the original scale, and after discussion by the research group, a Chinese version was formed.2 ③Cross-cultural adaptation: according to the principle of cultural adaptation [17], seven experts (three in the field of clinical nursing, three in the field of nursing education, and two in the field of nursing psychology) with rich scientific research experience were invited to evaluate the applicability, relevance, and completeness of the semantics, criteria, and concepts of the Chinese version of the scales, and to modify and form the Chinese version based on the experts’ opinions.3 The Chinese version of the scales was published in the Chinese Journal of Nursing, which was published by the National Nursing Association of China. ④ Pre-test: 20 nursing students were selected and pre-tested using the General Information Questionnaire and Chinese version 3. The results showed that the scale took about 5 min to complete and the content was understandable. Interviews were conducted with the respondents after the test, and the comments made by the respondents about the questionnaire instructions, each item and option were discussed and modified to adjust the content of the questionnaire and form the first draft of the final version of the Chinese version.

### Data collection procedure

The researchers collected questionnaires from three provinces. A total of 506 clinical nursing interns were recruited in hospitals using convenience sampling. 494 people volunteered to take part in a cross-sectional survey of these populations. All questionnaires were completed anonymously. After excluding invalid questionnaires, a total of 486 questionnaires were collected. The effective recovery rate was 98.38%.

### Data analysis procedure

The 14 items of the scale were ranked from lowest to highest total score, with the low group representing the 27% of the sample with the highest total score and the high group representing the 27% of the sample with the lowest total score, all subjected to independent samples t-tests. The reliability and discrimination of the translations were judged by comparing the relationship between the two. A critical rate was applied to see if there was a statistical significance among the different items. Cronbach’s alpha coefficient was used to determine whether the translated scale items needed to be deleted.

#### Reliability analysis

Reliability refers to the consistency or repeatability of a measurement [18]. The internal consistency of the scale was assessed using Cronbach’s a coefficient and folded half reliability. Items are divided in odd and even order, and the correlation between items is calculated to express the fold-half confidence. The interval between the two measurements for nursing students was 2 weeks. 2 weeks later, The scale was used to remeasure 40 clinical nursing students who were previously labeled, which reflects the consistency of the two tests.The Intraclass Correlation Coefficient (ICC) was calculated to assess intra- and interrater reliability of the scale.

#### Validity analysis

Seven eligible experts were invited to evaluate the content validity of the Chinese version of the scale. The content validity index of the items (I-CVI) and the content validity index of the scale (S-CVI) were calculated using the Lawshe evaluation method. Each item is divided into 4 levels (from no relevance to high relevance). The I-CVl value is equal to the ratio of the number of experts scoring 3 or 4 to the total number of experts involved in this evaluation. S-CVl is the average of I-CVl for all items. The potential factor structure of the scale was explored and validated using the exploratory factor analysis (EFA) and the confirmatory factor analysis(CFA), respectively. Based on the principle of randomization, 486 participants were divided into two groups with equal numbers in each group. The EFA and CFA tests were administered to 243 participants in each group. If the Bartlett sphere test had statistical significance when the Kaiser-Meyer-Olkin (KMO) measure of sampling adequacy was greater than 0.6 (*P* < 0.05), then it would be appropriate for factor analysis. Based on the characteristic value, the ANOVA and the visual examination of the debris map, the factors were obtained. Analysis of moment structure (AMOS) Validation of Factor Model for CFA.

### Ethical approval

All participants filled out an informed consent form, and the information from the questionnaires filled out by the participants will be protected from disclosure. The research has been approved by Jinzhou Medical University’s Ethics Committee, and will abide by its code of ethics (JZMULL2023008).

## Results

### Demographic information

A total of 486 participants met the criteria: 399 females (82.1%) and 87 males (17.9%). The percentage of nursing students with a college degree was 40.7% and 59.3% with a bachelor’s degree or higher. The percentage of students who liked nursing as a career was 76.5%, but nursing students were satisfied with clinical satisfaction, with only 15.2% satisfied with their current clinical practice environment. The rest of the demographics are shown in Table 1.

**Table 1:** General demography data (*n* = 486)

Items	Group	n	%
Age	< 20	62	12.8
	≥ 20	424	87.2
Sex	Male	87	17.9
	Female	399	82.1
Educational level	Junior college education	187	38.5
	Undergraduate education and above	299	61.5
Internship duration	< 6 momths	163	33.5
	≥ 6 momths	323	66.5
Site	Town	179	51.1
	Country	307	48.9
Only child	Yes	212	43.6
	No	274	56.4
Enjoy the nursing profession	Yes	372	76.5
	No	114	23.5
Clinical satisfaction	Dissatisfaction	239	49.2
	Ordinary	173	35.6
	Satisfaction	74	15.2

### Item analysis

A critical ratio > 3.000 indicates a higher discriminability of the items. In the present study, the critical ratio of 14 items ranged from 9.047 ~ 20.938, indicating better discriminability of the items. There was significant positive correlation among all items (*r* = 0. 612-0. 762, *P* < 0.01), which showed that there was a moderate relationship among all the items. When the individual entries are removed, the Cronbach’s factor for the translation scale is between 0.907 and 0.915, which is in the Cronbach alpha range (0.916), indicating that these 14 items could be retained.

### Reliability analysis

The Cronbach’s conversion factor is 0.916. The Cronbach’s coefficient for each dimension was between 0.768 and 0.894. The fold-half confidence of the scale was 0.867. In addition, 40 nursing students were randomly selected to be retested again 2 weeks later to obtain a retest reliability of 0.901. This shows that the conversion scale has a good reliability (Table 2).

**Table 2:** Reliability analysis for Chinese version of the nursing students’ rights awareness scale

The scale and its dimension	Cronbach’s Alpha	Split-half reliability	Test-retest reliability
The awareness of nursing students’ rights	0.916	0.867	0.901
The rights to be protected, cared, supported, and respected	0.768		
The rights to learn	0.796		
The rights to be recognized as a member of a nursing team	0.894		

### Validity analysis

#### Content validity analysis

A total of 7 specialists were employed to evaluate the effectiveness of the English Translation Inventory. I-CVI has a range of 0.857 to 1.000, S-ICV was 0.963. Therefore, the translation measure was of high quality and content validity in the scale.

#### Exploratory factor analysis

**Fig. 1:**
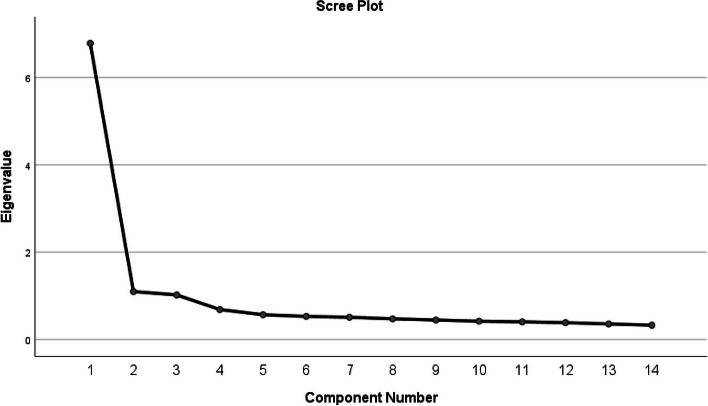
Screenshot of exploratory factor analysis of psychometric properties of the Chinese version of the Rights Awareness Scale for Nursing Students

In this trial, KMO = 0.946 and the Bartlett sphere were statistically significant (χ^2^ = 3198.060; *P* < 0.001), which were appropriate for the analysis of the factors [19]. Orthogonal rotation of the data was carried out by principal factor analysis and maximum variance, and the results showed that the values of the three parameters were higher than 1. The cumulative variance explained a total of 63.577% of the total variance. The presence of a three-factor structure was confirmed by the screenshot (Fig. 1). EFA results showed that each factor had a factor loading greater than 0.4. This way, all 14 items can be kept without removing them. The results of the factor loadings are shown in the Table 3.

**Table 3:** Factor loadings of exploratory factor analysis for the psychometric properties of nursing students’ rights awareness scale

Item	Factor1	Factor2	Factor3
1	**0.742**	0.163	0.213
2	**0.736**	0.262	0.211
3	**0.730**	0.308	0.297
4	**0.709**	0.231	0.231
5	**0.684**	0.150	0.211
6	**0.671**	0.278	0.157
7	**0.664**	0.199	0.239
8	**0.588**	0.273	0.245
9	0.241	**0.791**	0.155
10	0.249	**0.787**	0.206
11	0.318	**0.753**	0.165
12	0.284	0.176	**0.777**
13	0.355	0.173	**0.769**
14	0.193	0.178	**0.726**

#### Confirmatory factor analysis

**Fig. 2:**
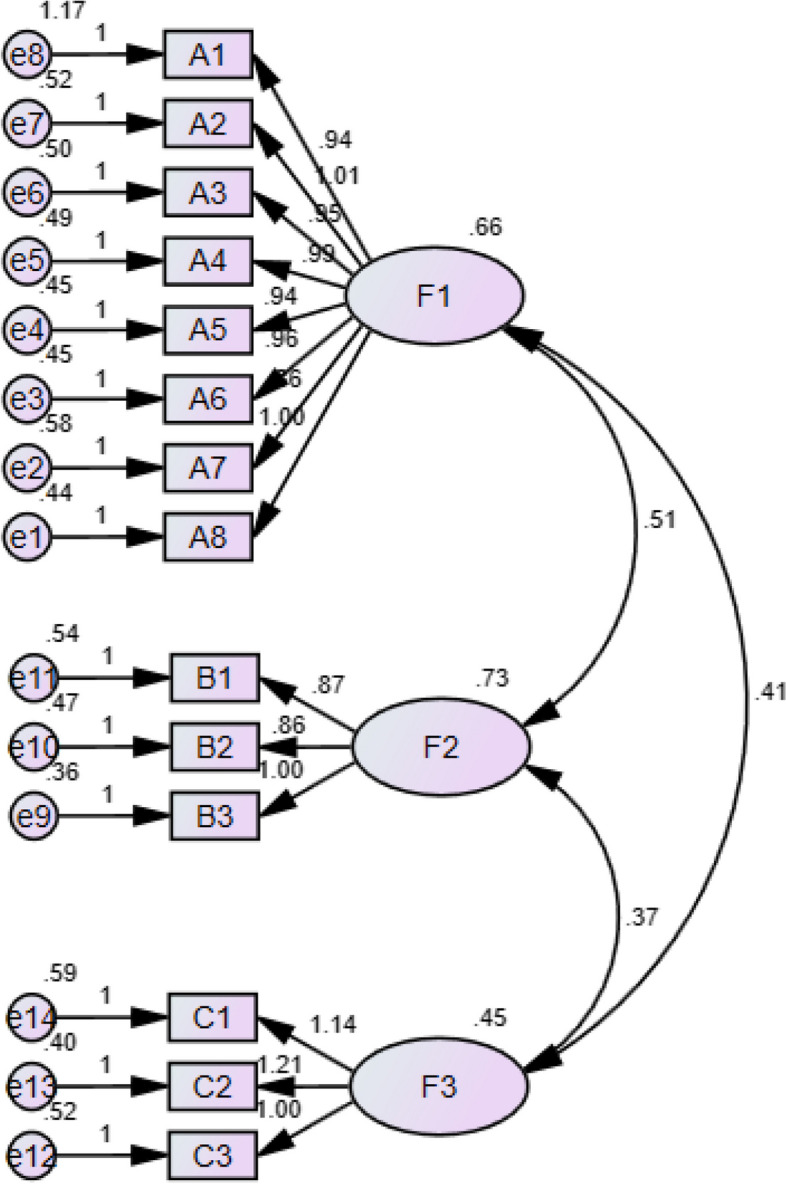
Standardized three-factor structural model of nursing students’ rights awareness scale (*n* = 486)

The results of the confirmatory factor analysis are shown in Fig. 2. In the final model fit index (original model fit index), each fitting index in the model fit index meets the statistical requirements, indicating that the overall fit of the model is better, and the results of each fitting index are shown in Table 4. The results showed that the modified scale factors corresponded well to the relevant items.

**Table 4:** Chinese version of the Rights Awareness Scale confirmatory factor analysis model fit indicators (*n* = 486)

Fit index	χ^2^/df	RMSEA	CFI	TLI	IFI	GFI
Refer to the standard specific value	1~3	< 0.08	> 0.9	> 0.9	> 0.9	> 0.9
	1.092	0.014	0.998	0.997	0.998	0.977

*RMSEA* is Root Mean Square of Approximation Error, *CFI* is Comparative Fit Index, *TLI* is Non-Gauge Likelihood Index, *IFI* is Value-Added Fit Index, *GFI* is Goodness-of-Fit Index

## Discussion

Nursing students studying in Chinese clinical practice settings lack instruments to assess their personal understanding of their rights. In this study, the original scale was translated into Chinese for the first time, and its psychometric properties were tested and confirmed in a clinical nursing student population by conducting factor analysis [20]. But clinical practice offers a chance for the nursing student to acquire implicit knowledge, In addition, it can be used as an important strategy for nursing educators to improve nursing students’ understanding of their rights in clinical practice, and it can be effective in increasing nursing students’ confidence in performing clinical activities and improving the quality of nursing services while improving their knowledge and practice skills.

### Chinese version of the scale has a good degree of discrimination

The critical proportion of items in this research was 9.047 to 20.938, which were better than the standard values [21]. There was a moderate to high positive correlation between item scores and the overall score. The Cronbach’s alpha coefficient after deleting each item did not exceed the initial value of the translated scale, so there was no need to delete any items.

### Chinese scale has good validity

Validity refers to the degree to which the tool under test accurately corresponds to the real world. The validity of the scale was evaluated by content analysis and structural analysis. The Delphi method shows that both I-CVI and S-CVI are higher than the standard values [22]. The cumulative variance contribution rate of common factors was > 40%, and the corresponding factor load value of each item was > 0.4. The cumulative variance contribution rate of the three common factors of the English scale was 63.3%. In this study, three factors were extracted from the exploratory factor analysis, and the cumulative variance contribution rate was 63.577%, indicating that the structure of the Chinese scale was consistent with the original scale, and the three dimensions of the original scale were retained. The results of confirmatory factor analysis confirmed that the model fit of the Chinese version of the scale of nursing students’ awareness of rights in clinical practice was good, and all indicators were better than the original version [23]. Therefore, the Chinese version of the scale has ideal validity in clinical nursing students.

### Chinese scale has good reliability

Reliability can reflect the reliability and stability of the scale [24]. We analyzed the reliability of the Chinese version of the scale from three aspects: Cronbach’s α coefficient, broken half reliability and retest reliability. The results showed that the Cronbach’s a coefficient of the translation scale was 0.916, and the Cronbach’s α coefficient of each dimension was 0.768 ~ 0.894, which was basically consistent with the original scale, indicating that the scale had good internal consistency. The broken half reliability and retest reliability are 0.867 and 0.901 respectively, which indicates that the scale has good reliability and high stability. Therefore, the Chinese version of the scale can be used to assess the consciousness of rights of clinical nursing students.

### Limitations

This research has some shortcomings which should be taken into account and discussed. Firstly, there was no investigation on the influential factors of the nursing student’ rights consciousness, which is important for our future research. In addition, there are limitations in the geographical location of the subjects selected for this study, and it is recommended to expand the scope of the sample size to verify the applicability of this scale in a deep and extensive manner.

## Conclusions

The research indicates that the Chinese Rights Awareness Scale for Nursing Students in Clinical Practice is reliable and effective,which can be applied to evaluate the degree of rights awareness of clinical nursing students. The scale provides a basis for guiding and improving nursing students’ rights awareness and fighting for their rights in clinical practice.

### Implications

Nursing student rights awareness is particularly important as it relates to the future employability and motivation of nursing students and also contributes to the establishment of a supportive clinical study environment for nursing students and to improve their skills and quality of care for patients in accordance with their rights. Therefore, it is of great interest to assess the level of nursing students’ rights awareness.

## Data Availability

The data garnered during the current study and the final dataset used for statistical analysis are available from the corresponding author on reasonable request. The Chinese version of the Awareness of Nursing Students’ Rights in Clinical Practice Scale are available from the corresponding author on reasonable request.

## References

[1] Huang ZW, Zeng LF. Cultivation of nursing students’ awareness of rights protection and self-discipline. J First Mil Med Univ. 2005,28(01):18–9. Chinese.

[2] Guo SX. Cultivation of Civic Consciousness and Transformation of Thinking Mode [J]. Theory Modernization. 2010;0(6):101–5. 10.3969/j.issn.1003-1502.2010.06.019. Chinese.

[3] Kangasniemi M, Viitalähde K, Porkka S (2010). A theoretical study of nurses’ rights. Nurs Ethics.

[4] Günay U, Kılınç G (2018). Transfer of theoretical knowledge to clinical practice by nursing students and the difficulties they encounter: a qualitative study. Nurse Educ Today.

[5] Baraz S, Memarian R, Vanaki Z (2015). Learning challenges of nursing students in clinical settings. A qualitative study in Iran. J Educ Health Promot.

[6] Huang L. Status Investigation and Analysis of Clinical Study Environment of Nursing Interns. Heilongjiang Sci. 2020;11(21):160–1. 10.3969/j.issn.1674-8646.2020.21.076. Chinese.

[7] Campbell J. Guiltily. Chest. 2012;142(2):536. 10.1378/chest.11-2515.

[8] Kim SJ, Kim EJ (2017). An exploratory study on students’ rights in social work field internships: in the context of the right to learn and the right to work. Korean J Soc Welf.

[9] Gillespie M (2002). Student-faculty connections in clinical nursing education. J Adv Nurs.

[10] Huang YX, Mu SY, Zhu JL, et al. Clinical Practice Environment and Transition Shock among Nursing Students: The Mediating Role of Resilience.[J]. Nurs J Chinese People’s Laberation Army. 2021;38(11):8–11. 10.3969/j.issn.1008-9993.2021.11.003. Chinese.

[11] Papp I, Markkanen M, von Bonsdorff M (2003). Clinical environment as a learning environment: student nurses' perceptions concerning clinical learning experiences. Nurse Educ Today.

[12] Eastaugh SR (2012). The impact of health information technology on productivity. J Health Care Financ.

[13] Wiechula R (2016). General review of the evidence: what factors influence the caring relationship between nurses and patients?. J Adv Nurs.

[14] Park SH, Choi MY (2021). Development and validation of a rights awareness scale for nursing students in clinical practice: a Scale Development Study. Healthcare (Basel Switzerland).

[15] Wolf EJ, Harrington KM, Clark SL, Miller MW (2013). Sample size requirements for structural equation models. An assessment of power, bias, and solutions. Educ Psychol Meas.

[16] Bijani M, Mohammadi F, Haghani F (2021). Development and psychometric evaluation of a reflection on clinical practice questionnaire for nursing students. BMC Nurs.

[17] Beaton DE (2000). Guidelines for the process of cross-cultural adaptation of self-report measures. Spine.

[18] Bruton A, Conway JH, Holgate ST (2000). Reliability: what is it and how do you measure it?. Physiotherapy.

[19] Schreiber JB (2021). Issues and recommendations for exploratory factor analysis and principal component analysis. Res Soc Adm Pharm.

[20] Schreiber JB (2021). Issues and recommendations for exploratory factor analysis and principal component analysis. Res Soc Adm Pharm.

[21] Huang F, Wang H, Wang Z (2020). Psychometric properties of the perceived stress scale in a community sample of Chinese. BMC Psychiatry.

[22] Almanasreh E, Moles R, Chen TF (2019). Evaluation of methods used for estimating content validity. Res Soc Adm Pharm.

[23] Zhang ZH (2017). Structural equation modeling in the context of clinical research. Ann Transl Med.

[24] Koo TK, Li MY (2016). A Guideline of selecting and reporting Intraclass correlation coefficients for reliability research. J Chiropr Med.

